# Arginine requirement for egg production in Japanese quail

**DOI:** 10.1016/j.psj.2022.101841

**Published:** 2022-03-11

**Authors:** Michele Bernardino de Lima, Manoela Garcia Borgi Lino de Sousa, Anna Raísa Teixeira Minussi, Lizia Cordeiro de Carvalho, Aline Guedes Veras, Euclides Braga Malheiros, Edney Pereira da Silva

**Affiliations:** Department of Animal Science, Universidade Estadual Paulista, College of Agriculture and Veterinary Sciences, Campus de Jaboticabal, SP 14884-900, Brazil

**Keywords:** amino acid, Coturnix coturnix japonica, egg production, broken line linear-quadratic-plateau, requirement

## Abstract

The objective of this study was to determine the ideal arginine intake for egg production in Japanese quail using the dilution technique. A completely random design was used, with 8 treatments (seven concentrations of arginine plus a control diet) and ten replicates, totaling 80 Japanese quails. The digestible arginine levels included in the study were 0.361%, 0.603%, 0.843%, 1.084%, 1.204%, 1.311%, and 1.460%. The variables analyzed were feed intake, egg production (**EP**), egg weight, egg output (**EO**), feed conversion ratio, and body weight were performed using a mixed model. When the effect of arginine levels (*P* ≤ 0.05) was detected, the model's broken line linear-plateau (**BL**), quadratic-plateau (**BLq**), and the first intercept of the BLq in the plateau of BL were adjusted to determine the ideal arginine intake. It observed that the arginine levels modified the quail responses (*P* < 0.001). Egg production was 10% with the 0.361% arginine in diet and recovered (97%) with the 1.311% arginine diet. The BL and BLq models estimated 232 mg/quail per day and 351 mg/quail per day for EO, respectively. The first intercept obtained was 290 mg/quail per day or 1,411%, which was considered the optimum level arginine intake for EO in Japanese quail.

## INTRODUCTION

The essentiality of arginine for birds was addressed in a study by Arnold et al. (1936). Two subsequent studies showed that birds could not synthesize arginine from ornithine in the diet (Klose et al., 1938), only from citrulline (Klose and Almquist, 1940). Even in the presence of arginase, which is absent in the liver of birds (Krebs and Henseleit, 1932), the ornithine cycle described by Krebs and Henseleit (1932) is inoperative owing to their inability to convert ornithine into citrulline. In mammals, the three main enzymes involved in the intestinal synthesis of citrulline are proline oxidase, N-acetylglutamate synthase, and pyrroline-5-carboxylate (**P5C**) synthase (Wu and Morris, 1998; Wu et al., 2009). P5C synthase is absent in bird enterocytes (Flynn and Wu, 1996; Wu and Morris, 1998; Wu et al., 2009); therefore, citrulline synthesis cannot occur, making arginine essential. Thus, higher arginine concentrations are required in poultry diets (Fernandes and Murakami, 2010; Silva et al., 2012).

In diets based on corn and soybean meal, arginine is the fifth limiting amino acid, preceding the amino acids methionine+cystine, lysine, threonine, and valine (Santos, 2013). Despite the importance of amino acids, studies on the arginine requirement for birds in laying phase, for example commercial laying hens, breeders or laying quails, are too few or inadequate to consolidate specific understanding of egg production (EP). Thus, generalizations have been extracted from studies with growing birds (Ball et al., 2007).

Eight studies were found on the arginine requirement for Japanese quails in laying phase (Reis et al., 2012; Cavalcante, 2013; Santos, 2013; Silva, 2014; Bülbül et al., 2015; Maurício et al., 2016; Maurício et al., 2018; Tuesta et al., 2018) and the arginine requirement ranged from 0.945% to 1.472% or 253 to 377 mg/ bird per day. On the one hand, Ball et al. (2007) related that the publications were scarce, on the other hand, these publications have reported the absence of any effect of arginine on EP in Japanese quails. Nevertheless, each publication recommended the minimum dose studied.

The reported absence of any effects from arginine (Maurício et al., 2016; Maurício et al., 2018; Tuesta et al., 2018) does not allow analysis of the responses by the birds after being subjected to different intakes. Moreover, it is not possible to determine the degree of dietary limitation of arginine. It is essential to study the effects of arginine levels with greater amplitudes, especially for levels below 0.945%, to obtain an intake lower than 262 mg/bird perꞏd (Santos, 2013). Thus, we can reduce the responses of zootechnical performance, confirming that arginine is the first limiting amino acid in dietary protein. The dilution technique might be suitable to increase the range of arginine levels tested (Lima et al., 2020), using the dilution technique, obtained a 333% difference between arginine levels (0.237% and 1.027%) and confirmed its dietary limitation. The authors found a reduced EP by 50% and they could establish the ideal intake of arginine from the responses of the birds to various arginine levels. Therefore, the objective of the present study was to determine the ideal intake of arginine for EP in Japanese quails using the dilution technique.

## MATERIALS AND METHODS

### Animals, Housing, and Experimental Design

The present study was performed using Japanese quails. The Animal Ethics and Welfare Committee of Universidade Estadual Paulista approved all experimental procedures used in the study under protocol number 012203/17.

A total of 80 VICAMI Japanese quails (*Coturnix coturnix japonica*) at 22 wk of age with peak EP (97.4 ± 1%) and average body weight (191 ± 2 g) were used in the present study. The birds were housed individually in an open shed with galvanized wire cages measuring 0.40 m × 0.32 m × 0.15 m, which comprised the experimental units of the study. Nipple-type drinkers and trough feeders were used, modified with partitions to allow measurement per experimental unit. A 16-h photoperiod was used, with water and feed provided ad libitum. The design used was completely randomized, with eight treatments and ten replicates of one bird in each experimental unit.

### Experimental Diets

Two diets were formulated (Table 1), one with high protein content (L7, summit diet), but limited in arginine, meeting the requirements of energy and other nutrients. The second diet was formulated to be protein-free (L0), with the same nutritional levels as L7, except for amino acids and crude protein, according to Fisher and Morris (1970). The other treatments were obtained by dilution of the L7 diet, using the L0 feed as a diluent. Diets L0 and L7 were formulated to contain 0.0% and 1,512% of digestible arginine, respectively and after analysis, the contained in their composition were 0.0% and 1,460% of digestible arginine, respectively. According to Rostagno et al. (2011) the arginine requirement for quals is 1,213%. The proportions used in the dilution (Table 2) were enough to obtain the increasing arginine levels, while meeting the recommendations of the other nutrients according to the recommendations of Rostagno et al. (2011).

**Table 1:** Composition (g/kg) of the high protein (summit diet) and nitrogen-free (N-free) diets used in the arginine assay.

Item	Summit		N-Free	
Grain Corn	433.644		0.000	
Soybean Meal	372.421		0.000	
Corn Gluten 60% of CP	42.908		0.000	
Soy oil	55.000		25.000	
Wheat bran	0.249		0.000	
Dicalcium phosphate	12.106		17.459	
Limestone	68.085		68.229	
Salt	3.586		3.904	
Potassium chloride	0.000		13.384	
DL-Methionine 98	4.789		0.000	
L-Lysine 78	3.112		0.000	
L-Tryptophan 98	0.341		0.000	
L-Arginine 98	0.537		0.000	
L-Glycine 98	0.250		0.000	
L-Phenylalanine 98	1.443		0.000	
Rice husk	0.000		101.032	
Choline Chloride 60%	0.530		3.409	
Starch	0.000		516.582	
Sugar	0.000		250.000	
Vitamin and Mineral Premix^a^	1.000		1.000	

Total	1000.000		1000.000	

	Calculated	Analyzed	Calculated	Analyzed
AMEn^b^	3,000	-	3,000	-
Crude fiber	2.874	-	3.857	-
Potassium	0.833	-	0.700	-
Sodium	0.155	-	0.155	-
Calcium	3.000	2.950	3.000	2.943
Pd	0.323	0.318	0.323	0.315
Choline	1500	-	1500	-
Linoleic acid	4.023	-	1.315	-
Crude protein	24.57	25.58	-	0.150
Lysine	1.463	1.344	0.000	0.000
Methionine	0.828	0.748	0.000	0.000
Methionine+Cystine	1.200	1.139	0.000	0.000
Threonine	0.883	0.908	0.000	0.000
Tryptophan	0.308	0.292	0.000	0.000
Arginine	1.512	1.460	0.000	0.000
Valine	1.117	1.050	0.000	0.000
Isoleucine	1.024	0.961	0.000	0.000

a Content (per kg of the diet) – vit A, 7.000 IU; vit D3, 2.000 IU; vit E, 8 IU; vit K3, 2 mg; vit B1, 1 mg, vit B2, 3.5 mg; vit B6, 2 mg; vit B12, 5 mcg/kg; niacin, 25 mg; chlorine, 0.26 g; pantothenate acid, 10 mg; copper, 8 mg/kg; iron, 50 g; manganese, 70 g; zinc, 50 g; iodine, 1.2 mg and selenium 0.2 mg.

b AMEn: Nitrogen-corrected apparent metabolizable energy.

**Table 2:** Proportions of the summit and nitrogen-free diets (%), and analysed concentrations of the limiting arginine in Japanese quail diets.

Diets	Levels							
	L7	L6	L5	L4	L3	L2	L1	L8^a^
Summit (%)	100.00	91.65	83.33	75.02	58.33	41.70	25.00	25.00
Dilution (%)		8.35	16.67	24.98	41.67	58.30	75.00	75.00
L -Arginine 98% (g/kg)								0.258
Digestible Arginine levels in diets (%)	1,460	1,338	1,216	1,084	0.843	0.603	0.361	0.619^a^

a Control diet: a total of 2.58 g L-arginine 98% was added in the diet with lower amino acid level (L1) up to the second amino acid level in the diet (L2).

The treatments consisted of seven increasing arginine levels, plus a control diet. The experimental levels were L1: 0.361%, L2: 0.603%, L3: 0.843%, L4: 1,084%, L5: 1,204%, L6: 1,311%, and L7: 1,460% digestible arginine. The levels were selected considering the description of the response curve of quails such as initial (maintenance), linear and response stability phases (without additional response).

The eighth treatment, or control diet (L8), was used to prove the limitation of arginine as the first limiting amino acid in dietary protein. To confirm that the response of the birds was in function of the arginine, a control diet was included in the assay (L8). A small quantity of the crystalline amino acid (L-arginine 98%) was added to the diet with the lowest level of the amino acid (L1) sufficient to meet the level of the amino acid in the second-lowest level (L2). The amount of L-arginine supplemented was 0.26 g/kg in the control diet. An additional response (egg production, egg weight, egg mass) of birds subjected to diet L8 is expect when compared to that of the first level to prove the limitation of arginine.

Total amino acids content of the corn, soybean meal, and corn gluten 60% used in the formulation were analyzed using a Near infrared spectrometer by Evonik Industries, Guarulhos, São Paulo, Brazil. The total amino acid content in experimental diets were analyzed by CBO Laboratory, Valinhos, São Paulo, Brazil, using high-performance liquid chromatography (HPLC), and values obtained were corrected for digestible amino acids according to Brazilian Tables for Poultry and Swine (Rostagno et al., 2011).

### Experimental Procedures and Variables Analyzed

The assay lasted 8 wk, with the last 4 wk involving data collection, according to the guidelines set by Sarcinelli et al. (2020). Measurements of room temperature, EP, and daily mortality were collected. On 3 consecutive days in each week, the eggs were weighed to obtain the average weight, except for the first 2 levels and for the control diet, which were measured from the EP. This adjustment was necessary due to the reduction in EP in these treatments. Bird weighing was undertaken at the beginning and end of the assay, whereas feed leftovers were evaluated every 2 wk. A 15-d interval was established to quantify the leftovers, based on observations of behavioral patterns of Japanese quails in laying phase. A recent study by Silva et al. (2020) reported that the birds responded appreciably after experimental manipulation; therefore, a longer interval was considered between the measurements of feed leftovers. The mean temperature and relative humidity throughout the experimental period were 27°C and 36%, respectively.

The variables evaluated were feed intake (FI, g/bird per d), arginine intake (ARG_Intake,_ mg/bird per d), body weight (BW, g), body weight modification (ChangeBW, g/bird), EP (%/bird per d), egg weight (EW, g), egg output (EO, g/bird per d), and feed conversion by egg output (FCR, g/g), corrected for mortality.

### Statistical Analysis

The data were analyzed for the assumptions of homoscedasticity of variance (Brown-Forsythe) and normality of errors (Cramér–von Mises) using a mixed model. The difference between treatments 1 and 8, analysis of variance was performed. The experimental unit was considered as the random effect and the experimental arginine levels as the fixed effect. The variables were subjected to orthogonal polynomial contrast analysis for linear and quadratic effects of arginine levels. Considering that the levels were not equally spaced, the coefficients were generated by the correlation matrix using the procedure PROC IML by SAS.

When an effect was detected, considering a significance of 0.05 (*P* ≤ 0.05), regression analysis was applied using the broken line linear-plateau (**BL**) and quadratic-plateau models (Robbins et al., 2006). The value in the abscissa corresponding to the first intercept of the quadratic curve of the linear-plateau model was also calculated to determine the ideal arginine intake.

The adjusted models were:

Broken line linear-plateau (BL):

(Eq 1) γ=φ+α[λ−δ]

Where, (λ-δ) is 0 for δ values > λ, γ is the response, δ is the regressor variable, φ is the corresponding response to λ on the orderly axis, λ is the point that indicates a change in the trajectory of γ corresponding to the axis of the abscissas, and α is the slope.

Broken line quadratic-plateau (**BLq**):

(Eq 2) $$\gamma \;=\;\;\xi \;\;+\;\;\beta \;{\left[\tau \;-\delta \right]}^{2}$$

Where, (τ - δ) is 0 for δ values > τ, γ is the response, δ is the regressor variable, ξ is the corresponding response to τ on the orderly axis, τ is the point that indicates a change in the trajectory of γ corresponding to the axis of the abscissas, and β is the slope.

The models were adjusted using arginine intake (mg/bird per day) and arginine concentration (**ARG_c_**) as a percentage in the diet, with the *n* regressor variable. The value in the abscissa corresponding to the first intercept of the quadratic-plateau curve on the plateau of the linear-plateau model (first intercept of BLq on plateau BL), was calculated as follows:

First intercept of BLq on the plateau BL:

(Eq 3) δ=−(−βλ+sqrt(βφ−βλ))/β

Statistical analyses and estimation of model parameters were performed using SAS 9.4 (Statistical Analysis for Windows, SAS Institute Inc., Cary, NC; 2014).

## RESULTS

### Arginine as the First Dietary Limiting

The results obtained between treatments L1 and L8, 0.361% vs. 0.619% of digestible arginine in the diet (Table 3) showed a significant effect on EW (*P* = 0.028), indicating that the birds responded to the L-arginine 98% supplementation in treatment L1. Other variables such as EO, FCR, and BW presented average values, which suggested that the responses had improved with L-arginine 98% supplementation. However, the experimental variability was greater than the treatment variance.

**Table 3:** Validation of arginine limitation in dietary protein (lowest amount of arginine in diet - L1 vs. control diet - L8) on responses of the daily feed intake, daily amino acid intake, egg production, egg weight, egg output, feed conversion ratio, mean body weight and change in body weight of Japanese quails from 22 to 30 weeks of age.

Levels	Feed intake(g/bird per day)	Amino acid intake (mg/bird)	Egg production(%)	Egg weight(g)	Egg output(g/day)	Feed conversionRatio (g/g)	Mean body weight (g)	Change in body weight (g)
0.361 - L1	17.2	65.1	10.3	8.2	0.86	20.02	161.2	−53.0
0.619 - L8^a^	18.1	114.2	11.9	8.8	1.04	19.89	169.6	−51.5
SEM	0.3	5.8	1.0	0.1	0.1	1.4	2.3	3.9
DF	18	18	18	18	18	17	18	18
*p*‐Value	0.139	<.0001	0.424	0.028	0.293	0.963	0.070	0.851

a Control diet: A total of 2.58 g L-arginine 98% was added in the diet with lower amino acid level (L1). SEM: standard error of the mean.

### Productive Performance

Productive performance responses were significantly affected by treatments (*P* < 0.01; Table 4). Based on the results of the contrast analysis, there was a significant difference in the linear and quadratic effects for the dietary arginine levels used in the present study.

**Table 4:** Average responses to treatments for daily feed intake, daily amino acid intake, egg weight, egg output, feed conversion ratio, final body weight and change in body weight of Japanese quails from 22 to 30 weeks of age.

Levels	Feed intake(g/bird per day)	Amino acid Intake(mg/bird)	Egg production(%)	Egg weight(g)	Egg output(g/day)	Feed conversionratio (g/g)	Final body weight (g)	Change in body weight (g)
0.361 - L1	17.2	62.1	10.3	8.2	0.86	20.02	134.7	−53.0
0.603 - L2	20.5	123.3	50.6	9.0	4.5	5.02	154.8	−34.4
0.843 - L3	22.6	190.2	72.0	9.9	7.13	3.28	165.4	−25.5
1.084 - L4	24.5	265.5	93.9	11.1	10.4	2.38	176.9	−12.4
1.204 - L5	25.2	303.0	96.0	11.7	11.2	2.26	195.9	1.2
1.311 - L6	24.9	323.9	97.1	11.7	11.4	2.19	195.7	1.0
1.460 - L7	24.8	358.4	94.1	11.4	10.8	2.33	189.0	3.5
SEM	0.4	12.6	3.9	0.2	0.5	0.8	3.0	2.8
*P*‐Value								
Treatment	<0.0001	<0.0001	<0.0001	<0.0001	<0.0001	<0.0001	<0.0001	<0.0001
Linear	<0.0001	<0.0001	<0.0001	<0.0001	<0.0001	<0.0001	<0.0001	<0.0001
Quadratic	<0.0001	<0.0001	<0.0001	<0.0001	<0.0001	<0.0001	0.0001	0.0003

L: Level; SEM: standard error of the mean.

Birds which consumed the lowest amount of arginine in diet (0.361%) had a reduced feed intake by 44%, whereas arginine intake was reduced by approximately 83% of the maximum intake given in mg/bird per day (83% = [1−62/358] × 100). Egg production (**EP**) decreased by 89% (89% = [1−10.3/97.1] × 100), which is proportional to the reduction in arginine intake. Egg weight (**EW**) was modified with the reduction in arginine intake. However, the reduction was close to 30%, in relation to the maximum response, and this variable presented the lowest reduction compared to the other production variables. Egg output (**EO**) relates to EP and weight. When considering the average of the lowest level of arginine, EO obtained the highest reduction, which was close to 92%. Thus, the lowest level of arginine was close to the region of maintaining the BW. FCR was modified to 89%, as well as EP, with an average value of 20.02 g/g to 0.361% of digestible arginine in the diet. Meanwhile, the birds fed with 1,311% had an average feed conversion of 2.19 g/g. The high values of FCR, especially for the 0.361% level of arginine were associated with a reduction in EP, and modification of BW and EW, in addition to reduced arginine intake. The birds in this treatment lost 53 g of BW during the entire experimental period. All variables were significantly correlated (*P* < 0.001), as shown in the value of *ρ* in Table 5 and showed a strong correlation value of *ρ* > 0.7.

**Table 5:** Correlation between responses for daily feed intake, daily amino acid intake, egg production, egg weight, egg output, feed conversion ratio, final body weight and body weight change of Japanese quails from 14 to 22 weeks of age.

Item	Feed intake(g/bird per day)	Arginine intake (mg/bird)	Egg production (%)	Egg weight(g)	Egg output(g/bird per day)	Feed conversionRatio (g/g)	Final body weight(g)	Body weightChange (g)
Feed intake	1	0.906	0.909	0.828	0.917	−0.750	0.836	0.807
Arginine intake		1	0.895	0.874	0.932	−0.710	0.826	0.850
Egg production			1	0.803	0.980	−0.849	0.775	0.790
Egg weight				1	0.895	−0.669	0.843	0.806
Egg output					1	−0.796	0.822	0.817
Feed conversion ratio						1	−0.639	−0.649
Body weight final							1	0.895
Body weight change								1

### Responses of Birds to Arginine Levels and Estimation of Optimal Intake

To interpret the linear and quadratic effects of arginine levels, the parameters of the linear-plateau and quadratic-plateau models were adjusted. The equations obtained presented r² values for all variables, higher than 0.7, for linear-plateau and quadratic-plateau models (Table 6).

**Table 6:** Estimated parameters of the linear and quadratic-plateau models for daily feed intake, egg production, egg weight and feed conversion ratio of Japanese quails from 22 to 30 weeks of age.

Variables (γ)	Unit (δ)	Broken line: linear- plateau (BL)						Broken line: quadratic- plateau (BLq)					
		φ	α	Λ	r²	BIC	*P*‐Value	ξ	β	τ	r²	BIC	*p*‐Value
FI	%	24.9	−9.944	1,102	0.81	244	<0.0001	25.0	−7.458	1,388	0.81	242	<.0001
	mg/bird per day	24.8	−0.045	232	0.85	226	<0.0001	25.3	0.000	365	0.87	218	<.0001
EP	%	97.6	−121.100	1,031	0.89	528	<0.0001	99.2	−88.500	1,347	0.89	526	<.0001
	mg/bird per day	95.5	−0.472	235	0.91	516	<0.0001	96.7	−0.001	320	0.91	510	<.0001
EW	%	11.6	−4.137	1,204	0.78	149	<0.0001	12.1	−1.787	1,871	0.76	158	<.0001
	mg/bird per day	11.7	−0.014	310	0.79	147	<0.0001	12.4	0.000	530	0.77	152	<.0001
FCR	%	2.9	100.130	0.532	0.86	316	<0.0001	2.5	115.350	0.751	0.88	305	<.0001
	mg/bird per day	2.9	0.462	100	0.85	323	<0.0001	2.7	0.003	142	0.86	315	<.0001

FI: Feed intake; EP: Egg production6; EW: egg weight; FCR: Feed conversion ratio; BIC: Bayesian Information Criterion.

r^2^: Coefficient of determination.

γ: response variable.

φ and ξ: maximum response.

α and β: is the slope.

Λ and τ: requirement for maximum response.

The quadratic-plateau models estimated τ values greater than λ of the linear-plateau for the same variable. The exception to this was for EW, which, although significant, had an estimated value for τ that was outside the range of 0.361% and 1,460% of digestible arginine in the diet (Table 6). In addition, according to the statistical adjustment (r²) and model selection (BIC), the selected model indicated that the linear-plateau model was the best choice.

The models, adjusted for feed conversion when regressed as a function of the percentage of arginine in the diet, presented a good adjusted for r² value. Even when δ was used in the formula, mg/bird perꞏd, the parameters adjusted for both models should be interpreted with limitations, especially for the biological meanings of λ and τ, which do not refer to the concentration and optimal arginine intake in the diet (Table 6). It is simply the point that indicates the change in the trajectory of γ corresponding to the axis of the abscissas.

**Figure 1:**
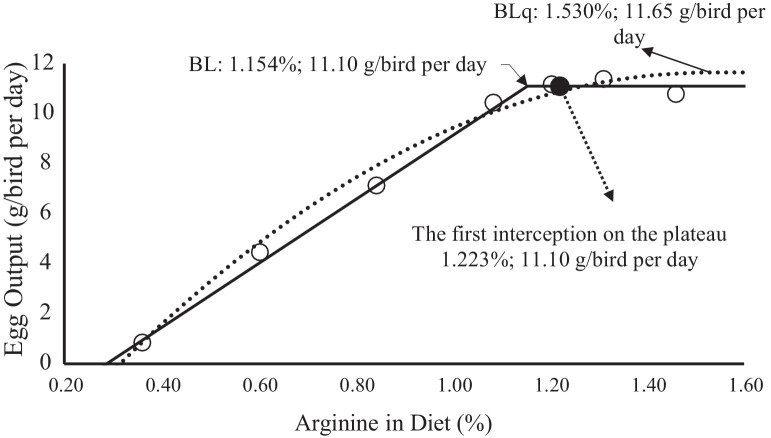
Relation between arginine in diet (%) and egg output (g/bird per day). ο -Observed values egg output. ——, Predicted values egg output by broken-line model (BL). ......., Predicted values egg output by quadratic broken-line model (BLq). •- Calculated value the first interception of BLq on the plateau of the BL.

**Figure 2:**
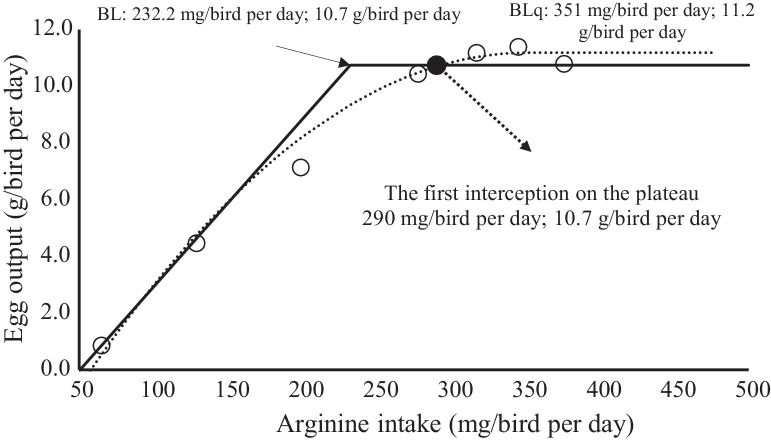
Relation between arginine intake (mg/bird per day) and egg output (g/bird per day). ο - Observed values egg output. ——, Predicted values egg output by broken-line model (BL). •, Predicted values egg output by quadratic broken-line model (BLq) ....... - Calculated value the first interception of BLq on the plateau of the BL.

EO presented the highest values of *ρ,* therefore, it was the variable selected to detail the interpretations of the effect of arginine on the Japanese quails in laying phase Figure 1, Figure 2. show the relationship between EO and arginine concentration in the diet (Figure 1) and arginine intake (Figure 2). In these figures, the prediction of the linear-plateau and quadratic-plateau models, versus the values of means observed in the treatments are plotted.

The linear-plateau (γ = 10.7(±0.2)–0.059 (±0.004) ×[232(±8) − δ], r² = 0.91, and BIC = 225) and quadratic-plateau models (γ = 11.2(±0.3)–0.0001 (±0.0000) ×[351(±14) − δ]², r² = 0.92, and BIC = 218) were adjusted following the constraints to form the plateau. Thus, when δ > λ was considered, (λ−δ) = 0 for the linear-plateau model and when δ > τ was considered, (τ−δ) = 0 for the quadratic-plateau model.

Even in the response by the birds to nutrient intake, the linear-plateau (γ = 11.1(±0.2) − 12.80 (±0.73) × [1,154(±0.034) – δ], r² = 0.92, and BIC = 222) and quadratic-plateau models (γ = 11.6(±0.4) –7.88 (±906) × [1,530(±0.077) − δ]², r² = 0.90, and BIC = 232) were adjusted considering the percentage of arginine in the diet. Similarly, the restrictions for plateau formation should be considered so that for δ > λ values, it is considered (λ−δ) = 0 for the linear-plateau model, and for δ > τ values, it is considered (τ−δ) = 0 for the quadratic-plateau model.

The estimated concentration and arginine intake corresponding to the first intercept of the quadratic-plateau curve in the plateau of the linear-plateau model was 1,210% or 302 mg/bird per d for feed intake, 1,164% and 205 mg/bird per d for EP, and 1,223% or 290 mg/birdꞏper d for EO (Table 7). EW and FCR estimates were not considered because they provided τ and λ values that were inconsistent for use as a recommendation.

**Table 7:** Arginine requirements according to the models for daily feed intake, egg production, egg weight, egg output and feed conversion ratio of Japanese quails from 22 to 30 weeks of age.

Variable	Arginine acid intake (mg/bird per day)			Concentration in the diet (%)		
	Broken-line model	Quadratic broken-line model	First interception of BLq on the plateau of the BL	Broken-line model	Quadratic broken-line model	First interception of BLq on the plateau of the BL
Feed intake	232 ± 11	365 ± 19	302	1.102 ± 0.043	1.388 ± 0.109	1.210
Egg production	235 ± 9	320 ± 19	205	1.031 ± 0.036	1.347 ± 0.052	1.164
Egg weight	310 ± 16	530 ±2 9	498	1.204 ± 0.001	1.871 ± 0.420	1.746
Egg output	232 ± 8	351 ± 14	290	1.154 ± 0.034	1.530 ± 0.077	1.223
Feed conversion ratio	100 ± 10	142 ± 9	91	0.532 ± 0.122	0.751 ± 0.035	0.473

## DISCUSSION

The main challenge in the present study was to describe the response curve of Japanese quails subjected to different concentrations of arginine, as there were no results found in the literature demonstrating a reduction in the responses of the birds, especially for the EP variable, via arginine deficiency in the diet. The results obtained in the present study demonstrated that arginine is essential for Japanese quails in laying phase, and its deficiency significantly affected EP, EW, EO, and BW.

When the dilution technique is used, it is necessary to prove the limitation of the amino acid studied (Sarcinelli et al., 2020; Silva et al., 2019). Thus, it is expected that the birds will also respond to L-amino acid supplementation to confirm the limitation generated. The results presented in Table 3 show that L-arginine supplementation significantly increased EW by 0.6 g (*P* < 0.05), which is approximately a 7.3% increase or 11.4 mg of egg for each mg of arginine. Even though the EO was higher in treatment L8, approximately 22% higher than that of L1, this increase could not be detected with the F test.

In the present study, analysis of variance (**ANOVA**) was applied between L1 and L8 to verify the statistical significance of the additional responses obtained with L-arginine supplementation. This was investigated due to reports of arginine having no effect on EP (Maurício et al., 2016; Maurício et al., 2018; Tuesta et al., 2018). Other studies that also used the dilution technique did not use this amount of rigor to validate the limitation of the amino acid test (Silva et al., 2019; Lima et al., 2020; Sarcinelli et al., 2020) due to the variability in the responses obtained with this degree of nutritional deficiency. For some categories, body reserve attenuates the additional response (Silva et al., 2015a; Lima et al., 2018); therefore, in previous studies only the numerical value of the biological response was considered as suitable to prove the dietary limitation of the amino acid test.

The effect seen on EW can be attributed to the ecological characteristics of the species (Bowmaker and Gous, 1991) in preserving embryo competitiveness and maintaining a minimum EW to ensure posthatch survival (Bowmaker and Gous, 1991; Silva et al., 2015a; Lima et al., 2018), with the slightest variation was detected as significant by ANOVA (Table 3). The EW reduction in broiler breeder hens is close to 10% (Silva et al., 2015b) and in commercial laying hens it is close to 20% (Silva et al., 2015a), whereas for Japanese quails in the present study, a value close to 30% was recorded (Table 4), which was similar to that reported by Sarcinelli et al. (2020). These results indicate that the hens used in zootechnical production utilized methods to maintain the weight of the egg within a strict range. Nevertheless, all these studies reported BW loss (Bowmaker and Gous, 1991; Silva et al., 2015a; Silva et al., 2015b; Lima et al., 2018; Sarcinelli et al., 2020; ) and it is believed that body reserve mobilization had occurred to meet the regulation of EW.

Table 4 shows the mean values of EP based on the experimental levels. The birds reduced EP with the increasing degree of arginine deficiency in the diet, diverging that the absence of response reported in the literature might be partly related to the use of narrow ranges of experimental levels (Maurício et al., 2016; Maurício et al., 2018; Tuesta et al., 2018) The present study presents the first supportive evidence that a reduction in arginine levels affects EP (Table 4).

The correlation obtained between EP and feed intake showed that these variables were strongly correlated (*ρ* = 0.909; *P* < 0.001) (Table 5). It also illustrated that EP was directly affected by feed intake, which decreased by 44% in the treatment with 0.361% arginine in the diet. However, this feed intake decrease might be related to the intake regulation and physical intake capacity, especially in birds subjected to the 0.361% arginine in the diet who by the end of the assay had lost approximately 28% of their BW. In addition, the strongly correlated between body weight change and arginine intake showed that lower arginine levels are related to higher body weight change (Table 5).

According to the model of Silva et al. (2020), to meet the maintenance and EO production of the quails fed with 0.361% of arginine in the diet, an intake of 179 kJ/bird perꞏd or 43 kcal/birdꞏper d would be sufficient. However, the birds ingested 216 kJ/birdꞏper d or 52 kcal/birdꞏper d, demonstrating that feed intake of 17.2 g/birdꞏper d was an attempt to increase arginine intake. This is clear when the expected feed intake is considered, obtained from the relationship between expected metabolizable energy intake and energy concentration in the experimental diet (3,000 kcal/kg). The expected feed intake was calculated to meet the maintenance and EO of treatments 0.361%, 0.603%, 0.843%, 1,084%, 1,204%, 1,311%, and 1,460%. Thus, feed intake of 14.2 g/birdꞏper d was expected at 18.6 g/bird perꞏd, 21.7 g/birdꞏper d, 25.4 g/bird perꞏd, 26.8 g/bird perꞏd, 27.0 g/birdꞏper d, and 25.7 g/birdꞏper d, respectively, for the treatments mentioned. In this perspective, the birds subjected to the first three treatments (0.361%, 0.603%, and 0.843%) increased feed intake by 21%, 10%, and 4%, respectively, in relation to the values expected to meet the productive level, and consequently, these birds ingested excess nutrients and energy. For the other treatments, there was a reduction in the current feed intake in relation to the expected values for the productive level, which was considered sustainable. Furthermore, it is in accordance with the theory of consumption regulation by the most limiting nutritional resource proposed by Emmans (1994) and recognized by Gous (2010), Sakomura et al. (2015), Silva et al. (2020).

The variables showed linear and quadratic effects for the various arginine levels. For the interpretation of these effects, the linear and quadratic models were adjusted with plateau formation. The quadratic-plateau adjustment for EW estimated a τ value at 1,871% of arginine in the diet, which is outside the tested range. As mentioned, the EW has a precise regulation and the rate of variation (Δmg/mg = γ_n_-γ_n-1_/δ_n_−δ_n-1_, was Δ_t2-t1_ = 13.1, Δ_t3-t2_ = 13.5, Δ_t4-t3_ = 15.9, Δ_t5-t4_ = 16.0, Δ_t6-t5_ = 0.00, and Δ_t7-t6_ = −8.7) increased linearly up to 1,204% and recommended an intake of 303 mg/birdꞏper day of arginine (Table 4), after the instantaneous derivative, which was 0 (Δ_t5-t6_ = 0.0 mg/mg) and reduced by 8.7 mg in the last range (Δ_t6-t7_). This abrupt cessation reinforces the precise regulation of the EW that was reasonably adjusted by the linear-plateau model with r² = 0.78, whereas the quadratic-plateau model required a higher number of decreasing rates, different from zero, to fit the dataset. For this reason, quadratic-plateau adjustment was not considered to establish a nutritional recommendation for arginine. Other studies have reported the overestimation of the quadratic polynomial model (Araújo et al., 2014; Silva et al., 2015b); however, in the present study, the specificity of the animal model might have prevailed. This specificity is clear when comparing the feed conversion value of the lowest level among laying birds, which for Japanese quails was 20.02 g/g, for broiler breeder hens, it was 4.94 g/g (Silva et al., 2015b), and for commercial laying hens, it was 4.71 g/g (Silva et al., 2015a). The value of 17.4 g/g obtained by Bendezu et al. (2015) with commercial laying hens was the approximation closest to that obtained in the present study. However, Bendezu et al. (2015) used a dilution of 85% at the lowest experimental level, resulting in 3.24% crude protein in the diet, while the crude protein value in the present study was almost double. Even then, the Japanese quails presented the highest values of feed conversion. It is possible to extract from these results that Japanese quails are more sensitive to nutritional deficiency, which could be related to the body reserve capacity and mobilization used to maintain the minimum EP. However, there are birds of broiler matrices that are less sensitive to nutritional deficiency and have greater capacity to mobilize body reserves to maintain EP. This hypothesis is supported by the study undertaken by Silva et al. (2019), who recorded the lowest EO of 0.18 g/birdꞏper d and feed conversion of 61 g/g. The study by Sarcinelli et al. (2020) also showed the sensitivity of Japanese quails, with an EO of 0.5 g/bird per d and feed conversion of 32 g/g.

The change in the rate of change (Δ_t2-t1_ = 0.059, Δ_t3-t2_ = 0.039, Δ_t4-t3_ = 0.043, Δ_t5-t4_ = 0.021, Δ_t6-t5_ = 0.010, and Δ_t7-t6_ = −0.017) was approximately exponential between the first 3 levels. This characteristic made it difficult to adjust the linear-plateau and quadratic-plateau models. One option would be to use segmented models; however, the breakpoint would coincide with the second treatment, modifying the breakpoint as a nutritional recommendation, and would continue to be interpreted as a point that indicates a change in the trajectory of γ corresponding to the axis of the abscissas. Therefore, it is recommended that future studies use exponential models to interpret feed conversion in studies using the dilution technique.

The option used in the present study considering the value calculated in the abscissa corresponding to the first intercept of the quadratic curve of the linear-plateau model as a recommendation for optimal arginine intake was based on the acceptance of this procedure by several researchers Baker et al. (2002), Robbins et al. (2006), Siqueira et al. (2009), Araújo et al. (2014), Bendezu et al. (2015), Silva et al. (2015b), and Lima et al. (2018).

Eight studies were found on arginine requirement for Japanese quails in laying and, according to the adjusted models, the stabilization of the responses of the birds started from an intake of 235 ± 9 mg/bird per d. Therefore, the lowest intakes tested by Cavalcante, 2013: 253 mg/bird per d; Tuesta et al., 2018: 259 mg/bird per d; and Santos, 2013: 262 mg/bird per d are the minimum amounts of arginine required to supply the EP. To meet the EO production, the minimum intake established by the linear-plateau model was 232 ± 8 mg/bird per d, which is within the range obtained by Reis et al., 2012: 289 mg/bird per d, Maurício et al., 2016: 302 mg/bird per d, and Silva, 2014: 315 mg/bird per d.

The figures show the fit of each model and exact recommendation point obtained by the first intercept of the quadratic curve in the linear model plateau. The recommendations by this procedure were intermediate to the linear-plateau and quadratic-plateau models, which is a comfortable factor for nutritionists, mainly due to the quality of raw material among other challenges that prevent using the minimum nutritional recommendation. The recommendation of digestible arginine for EO in Japanese quails was 290 mg/bird per d. This value was close to that found by Bülbül et al. (2015) and Maurício et al. (2018).

If the 351 mg/bird per d estimated by the quadratic-plateau setting is not recommended for producers who market egg shells, it might be an option for producers who market eggs with their shells removed. Future studies should evaluate higher arginine levels in the diet as a facilitator for egg processing. Although Bülbül et al. (2015) did not evaluate this factor, they found that excess arginine impaired the quality of the egg shell.

## CONCLUSIONS

The recommendation of digestible arginine for Japanese quails at peak egg production was 290 mg/bird per day.
